# Optical and electron microscopy study of laser-based intracellular molecule delivery using peptide-conjugated photodispersible gold nanoparticle agglomerates

**DOI:** 10.1186/s12951-015-0155-8

**Published:** 2016-01-08

**Authors:** Judith Krawinkel, Undine Richter, Maria Leilani Torres-Mapa, Martin Westermann, Lisa Gamrad, Christoph Rehbock, Stephan Barcikowski, Alexander Heisterkamp

**Affiliations:** Institute of Applied Optics, Friedrich-Schiller-University Jena, Fröbelstieg 1, 07743 Jena, Germany; Institute of Quantum Optics, Leibniz University Hannover, Welfengarten 1, 30167 Hannover, Germany; Electron Microscopy Centre, Friedrich-Schiller-University Jena, Ziegelmühlenweg 1, 07743 Jena, Germany; Technical Chemistry I, University of Duisburg-Essen and Center for NanoIntegration Duisburg-Essen CENIDE, Universitätsstraße 7, 45141 Essen, Germany; Excellence Cluster REBIRTH, Hannover, Germany

**Keywords:** Cell-penetrating peptides, Gold nanoparticles, Particle agglomerates, Endosomes, Laser-based release, Intracellular molecule delivery

## Abstract

**Background:**

Cell-penetrating peptides (CPPs) can act as carriers for therapeutic molecules such as drugs and genetic constructs for medical applications. The triggered release of the molecule into the cytoplasm can be crucial to its effective delivery. Hence, we implemented and characterized laser interaction with defined gold nanoparticle agglomerates conjugated to CPPs which enables efficient endosomal rupture and intracellular release of molecules transported.

**Results:**

Gold nanoparticles generated by pulsed laser ablation in liquid were conjugated with CPPs forming agglomerates and the intracellular release of molecules was triggered via pulsed laser irradiation ($\lambda$ = 532 nm, $\tau _{pulse}$ = 1 ns). The CPPs enhance the uptake of the agglomerates along with the cargo which can be co-incubated with the agglomerates. The interaction of incident laser light with gold nanoparticle agglomerates leads to heat deposition and field enhancement in the vicinity of the particles. This highly precise effect deagglomerates the nanoparticles and disrupts the enclosing endosomal membrane. Transmission electron microscopy images confirmed this rupture for radiant exposures of 25 mJ/cm$^{2}$ and above. Successful intracellular release was shown using the fluorescent dye calcein. For a radiant exposure of 35 mJ/cm$^{2}$ we found calcein delivery in 81 % of the treated cells while maintaining a high percentage of cell viability. Furthermore, cell proliferation and metabolic activity were not reduced 72 h after the treatment.

**Conclusion:**

CPPs trigger the uptake of the gold nanoparticle agglomerates via endocytosis and co-resident molecules in the endosomes are released by applying laser irradiation, preventing their intraendosomal degradation. Due to the highly localized effect, the cell membrane integrity is not affected. Therefore, this technique can be an efficient tool for spatially and temporally confined intracellular release. The utilization of specifically designed photodispersible gold nanoparticle agglomerates (65 nm) can open novel avenues in imaging and molecule delivery. Due to the induced deagglomeration the primary, small particles (~5 nm) are more likely to be removed from the body.

**Electronic supplementary material:**

The online version of this article (doi:10.1186/s12951-015-0155-8) contains supplementary material, which is available to authorized users.

## Background

In modern medicine, the efficient delivery of chemically synthesized molecules or genetic material into cells is highly desirable. The main barrier for the delivery of molecules is the cell membrane. This lipid bilayer prevents passive molecule transport into the cytosol. Different approaches to deliver foreign molecules are currently being developed [1–4].

Cell-penetrating peptides (CPPs) have been developed as efficient molecule carriers [5–7]. CPPs have several advantages over other methods such as high cell throughput, low toxicity at low concentrations and, particularly, they can deliver molecular cargoes to different cell types without damaging the cell membrane [8]. Depending on the chosen peptide, specific subcellular or selective cell targeting may be possible [9, 10]. CPPs either directly penetrate the cell membrane or follow the endocytic pathway [8, 11]. Once encapsulated in endosomes a triggered release is essential for the biological cargo to take effect [8, 12–16]. This escape can occur in different ways. The rupture of the endosomes can be self-triggered by changes of the pH-level [2, 14] or externally activated by (laser)light [17–19], radiation, temperature, magnetic fields or ultrasound [1, 20].

Another attractive approach is to use gold nanoparticles as a vehicle for molecular cargo due to its inert and stable properties. The interaction of lasers with gold nanoparticles (AuNPs) was investigated for different (medical) applications [21–24]. Depending on the chosen parameters, occurring effects may include heat deposition and field enhancement or their subsequent impact like e.g. protein denaturation, bubble formation and creation of a pressure wave to kill [25–30] or manipulate biological material [30–33]. AuNPs are used to transiently open the cellular membrane in order to deliver extracellular molecules [31–35]. Laser-particle-interactions were also applied to open different carriers like nanocages, liposomes or synthetic constructs containing nanoparticles [36–38]. In addition, particles were applied for theranostic applications to combine therapy and diagnosis [24, 39, 40].

A critical issue is the biocompatibility of nanoparticle agglomerates. Here previous studies report on the advantages of biodegradable agglomerates cross-linked by polymers which dissipate in a cellular environment, while the resulting small nanoparticles can be easily cleared from the body via the kidney [41, 42]. The utilization of this concept in combination with laser-induced deagglomeration, however, has not been previously examined. It should be noted that small and ultrasmall gold nanoparticles, released after deagglomeration, have been previously reported to be cytotoxic [43, 44]. However, a recent comparison of toxicological studies has shown that these effects are predominantly caused by unrealistically high surface doses [45] which will never be reached in potential biomedical applications. We could, in addition, verify that small laser-generated nanoparticles, very similar to those applied in this study, are highly biocompatible, as they were proven not to interfere with critical functional cell parameters like oocyte maturation, even though the particles were taken up by the cells [46].

**Fig. 1:**
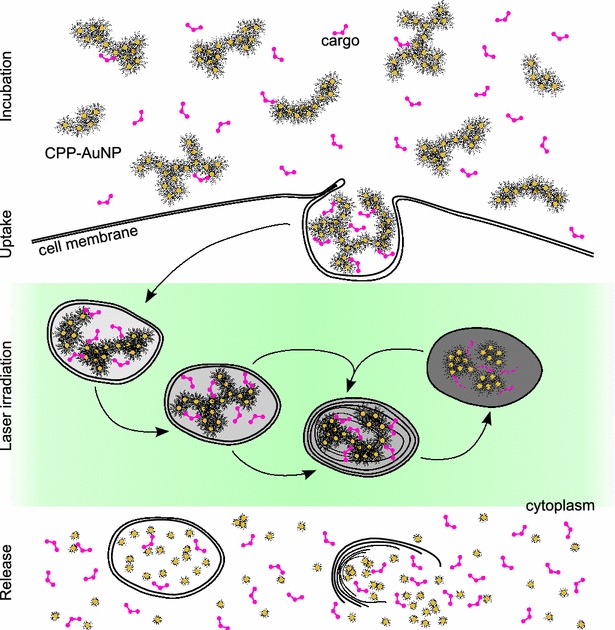
Schematic illustration of uptake and laser-based release of molecules co-incubated with CPP-AuNPs. During incubation CPP-AuNPs and the cargo are endocytosed. Inside the cell these endosomes mature unless their membranes are ruptured via laser irradiation to release the content to the cytosol

Combining both methods, CPPs in combination with controlled interaction of lasers and particle agglomerates, can lead to an efficient intracellular delivery of molecules without the need to disrupt the cell membrane. The larger agglomerate size provides better stimulus of the endocytic uptake, being assembled of smaller building blocks in a size range that is known to be more easily cleared via the kidney. Therefore, we conjugated AuNPs generated by pulsed laser ablation in liquid (PLAL) with CPPs resulting in deliberately agglomerated CPP-AuNPs. We recently characterized and studied the uptake of these agglomerated particle conjugates by mammalian cells [47]. In this study, we investigate the mechanisms of the targeted laser-induced release of intraendosomal molecules using a nanosecond pulsed laser and AuNP agglomerates. Figure 1 shows a schematic diagram of the process which was visualized using transmission electron microscopy (TEM) and fluorescence microscopy. Calcein molecules were utilized as cargo to be co-incubated with CPP-AuNP agglomerates, resulting in their concurrent uptake and presence in the endosomes. Laser irradiation of cells containing these endosomes ruptured the endosomal membrane and instantaneously released the deagglomerated particles and the cargo into the cytosol. We further studied the laser parameters necessary for an efficient molecule release and the viability of the cells after the treatment.

The combination of laser-triggered release and CPP-conjugated AuNP agglomerates has specific advantages. First, it is rather cell-type independent as the release mechanism is solely based on the interaction of the laser with the AuNPs and, therefore, depends on the parameters of those instead of the cell-type or the maturation stage of the endosomes. Second, the damaging effects are highly localized to the endosomal membrane as they are limited to the vicinity of the particles. This ensures that the treated cells retain normal functionality and unwanted damages of other cellular organelles are reduced. Additionally, the technique is spatially selective. Only laser-treated cells containing AuNPs exhibit endosomal rupture leaving other cells unaffected. And finally, the timing of the laser treatment can be arbitrary. One can temporally control the release of the molecules into the cytosol. Therefore, the presented technique is a promising method for a spatially and temporally specific delivery of chemically synthesized drugs or other molecules in mammalian cells. Furthermore, the facile renal clearance of the resulting deagglomerated small particles can ensure the biocompatibility of the presented method.

## Results

### Characteristics of CPP-AuNP agglomerates

**Fig. 2:**
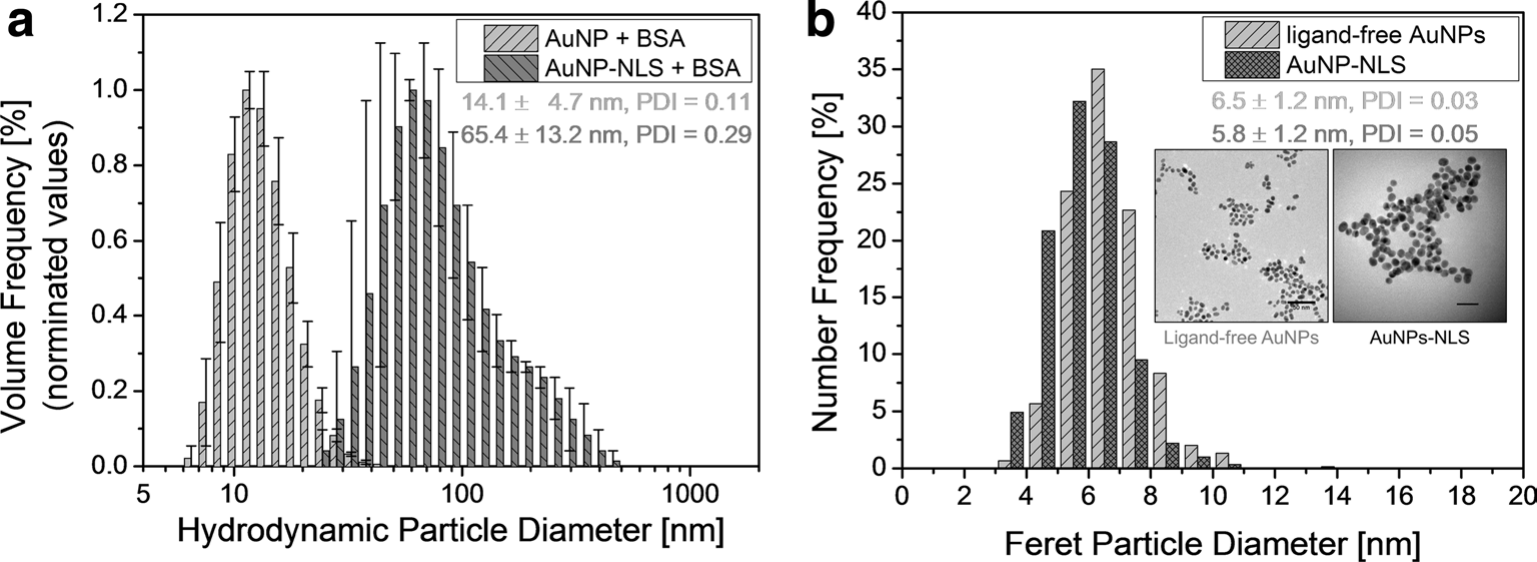
Characterization of ligand-free AuNPs and AuNP-CPP agglomerates. **a** Hydrodynamic diameter of ligand-free AuNPs and AuNP-NLS agglomerates (both stabilized with BSA) determined by dynamic light scattering. **b** Particle number distributions of agglomerated (NLS-conjugated) and primary, ligand-free AuNPs obtained from TEM-images. Representative images are depicted in the *insets* (*scale bars*: *left* 50 nm, *right* 20 nm)

CPP-AuNP agglomerates were prepared by using monodisperse laser-generated gold nanoparticles (see “Methods”) with a particle diameter of (5.0 $\pm$ 0.6) nm of the primary particles (see Additional file 1: Figure A4.1). Stabilized with BSA, which is a good stabilizer for nanoparticle-peptide-agglomerates and, additionally, is highly biocompatible, their hydrodynamic diameter is (14.1 $\pm$ 4.7) nm (Fig. 2a). The agglomerates are formed right after adding CPPs, deca-arginine or NLS, to the ligand-free primary particles. The agglomeration process is triggered by charge compensation between negatively charged AuNPs and positively charged peptides. This results in NLS-AuNP agglomerates (stabilized with BSA) with a hydrodynamic diameter of (65.4 $\pm$ 13.2) nm as deduced by dynamic light scattering (DLS) and a PDI of 0.29 (Fig. 2a). Data depicting their zeta potential of (+ 18.7 $\pm$ 14.9) mV as well as additional characteristics of deca-arginine agglomerates like the positive zeta potential of (+ 25.9 $\pm$ 5.0) mV and a shift of the surface plasmon resonance of 20 nm is shown in the Additional file 1 Section A2. Although strong agglomeration tendencies were verified, the characterization of the agglomerates by TEM shows the preservation of the primary particles (Fig. 2b) with a diameter of (5.8 $\pm$ 1.2) nm which is comparable to the diameter of ligand-free AuNPs of (6.5 $\pm$ 1.2) nm. Representative TEM-images are shown in the insets of Fig. 2b.

### Nanoparticles and their localization in the cells

**Fig. 3:**
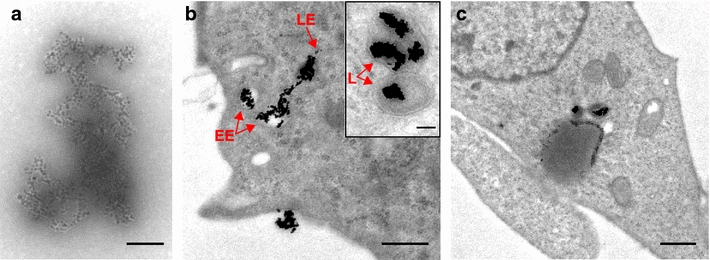
TEM-images of gold nanoparticles conjugated to CPP-AuNPs. CPP-AuNP-agglomerate in solution (**a**) and cells with CPP-AuNPs taken up via endocytosis (**b**, **c**). Endosomes with AuNPs can be found in different stages of maturation (**b**), *EE* early endosome, *LE* late endosome, *L* lysosome). **c** shows a more general overview of a cell containing endosomes with CPP-AuNPs. Scale bars: **a** 100 nm, **b** 500 nm, *inset* 100 nm, **c** 800 nm

TEM images of the CPP-conjugated negatively stained particle clusters show shape and size variations in the agglomerated state. The AuNP-clusters composed of strings of multiple particles that seem to be loosely attached to each other can reach sizes up to $\sim$550 nm in one dimension (Fig. 3a). Furthermore, the particles show a sheath of less electron dense material which we presume to be bound BSA as it can not be found in non-BSA stabilized agglomerates (Fig. 2b, both insets). This sheath can also be found in non-CPP-conjugated particle samples stabilized with BSA (Additional file 1: Figure A4.2a).

As shown in Fig.  3b, c, the CPP-AuNP agglomerates were endocytosed. We found particle-loaded endosomes all over the cytoplasm. In most cases, more than one agglomerate was found in a single endosome (Fig. 3b, inset). No particles were detected within the nucleus. Without laser-irradiation the agglomerates are retained inside the endosomes throughout their maturation stages (Fig. 3). Interpretation of the endosomal maturation stages in the TEM images follows the characteristics described in [48]. The agglomerates inside the endosomal compartments and agglomerates attached to the cell membrane appeared highly condensed compared to samples without cells (compare agglomerate in solution Fig. 3a to intraendosomal agglomerates 3c). Preferably in the later endosomal stages most of the agglomerates appeared more rounded up and showed less extensions.

### Effect of laser irradiation on particles and cells

**Fig. 4:**
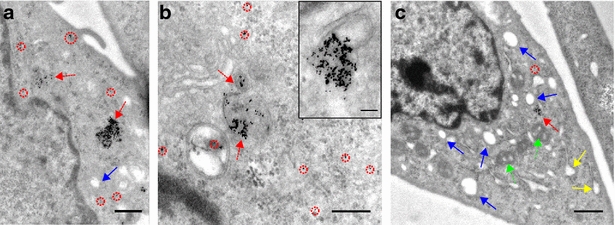
TEM images of laser-irradiated cells containing endosomes with CPP-AuNPs. Cells irradiated with 25 mJ/cm$^{2}$ (**a**) and 35 mJ/cm$^{2}$ (**b**, **c**). CPP-AuNPs are desagglomerated and endsosomes are partly or completely opened. Most particles are isolated, but still close to each other and the endosome they escaped (*red dashed arrows*). Some particles are distributed already inside the cytoplasm (*red dashed circles*). *Blue arrows* indicate laser-induced vacuoles. Vacuoles are also found in mitochondria (*green dotted arrows*) and blown up lumen of the endoplasmic reticulum (*yellow arrows*). *Scale bars*: **a** 500 nm, **b** 300 nm, *inset* 100 nm, **c** 800 nm

After irradiating particle agglomerates with a radiant exposure of 35 mJ/cm$^{2}$, the maximal radiant exposure used for the release study (see next section), no BSA-sheath was visible. Non-electron dense material with particle leftovers having a similar shape to the agglomerates was found (Additional file 1: Figure A4.1). In some of these structures, single nanoparticles were still present. Moreover, the agglomerates were mostly broken into isolated particles (Additional file 1: Figure A4.2b). Similarly, when AuNPs were endocytosed by the cells, laser irradiation induced separation of the particle agglomerates in the cells. Additionally, most of the endosomal membranes enclosing particles were fully or partially ruptured or completely dissolved (Fig. 4a,b). The inset in Fig. 4b is a typical image showing a partly ruptured endosomal membrane through which the particles enter the cytoplasm. Despite this strong effect no rupture of the outer cell membrane was observed. After irradiation isolated particles are found all over the cytoplasm (Fig. 4a–c, red dashed circles). The majority of the AuNPs are detached but still in the vicinity of the endosome they escaped from (Fig. 4a–c, red dashed arrows). Comparing cells irradiated with 25 mJ/cm$^{2}$ to cells treated with 35 mJ/cm$^{2}$, the latter ones showed distinctly higher amounts of vacuoles—areas showing no electron dense material (Fig. 4c, blue arrows). They might originate from blown up lumen of the endoplasmic reticulum (Fig. 4c, yellow arrows). Small vacuoles were also found in cell mitochondria (Fig. 4c, green dotted arrows). In cells irradiated with 25 mJ/cm$^{2}$ (Fig. 4a) this effect was rarely observed. Nevertheless, to a smaller extent such vacuoles were also observed in non-irradiated cells. A selection of further images of the intracellular particle release obtained by irradiation with 25 mJ/cm$^2$ compared to 35 mJ/cm$^2$ can be found in the Additional file 1: Figure A3.1 and Figure A3.2, respectively.

### Efficient calcein release into the cytoplasm

**Fig. 5:**
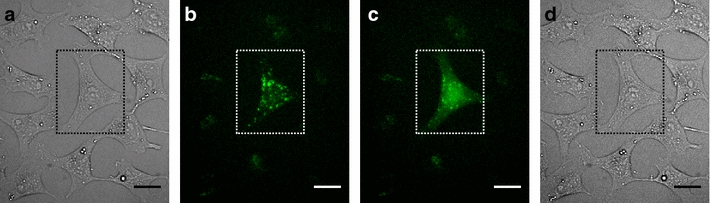
Visualization of calcein uptake and release. Calcein was co-incubated with CPP-AuNPs and cells for 4 h. The cell within the dotted box was irradiated. Brightfield images show no change of cell morphology after irradiation (**d**) compared to before (**a**). **b** Fluorescent image of cell before irradiation exhibits clear fluorescent spots. **c** After irradiation some of these spots are still visible, but calcein was released and spread all over the cytoplasm. *Scale bars*: 20 µm

4 h after co-incubating the cells with CPP-AuNP agglomerates and calcein, the cells contain small, localized and fluorescing spots (Fig. 5b). These are endosomes containing particle agglomerates and calcein. Irradiation of cells with these endosomes ruptures the endosomal membrane as confirmed with TEM (section above). The content diffuses throughout the whole cell leading to an overall increase of the fluorescing area (Fig. 5c) without a significant change on the cell morphology (Fig. 5a, d). The irradiation, however, does not completely dissipate the bright fluorescing spots. This indicates that not all endosomal content was completely released. Partly irradiated samples only show a visible release of dye into the cytosol in treated regions. In Fig. 5 only the cell inside the dotted box was irradiated. A selection of images showing different fields of view before and after irradiation can be found in the Additional file 1: Section A5, Figures A5.1 and A5.2.

**Fig. 6:**
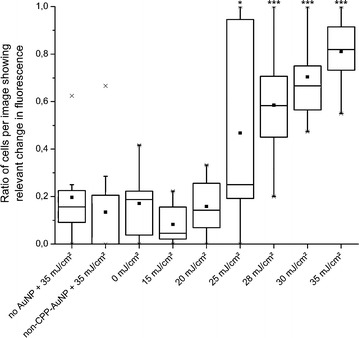
Evaluation of efficient intracellular release for different radiant exposures. Cells with endosomes containing CPP-AuNPs and calcein irradiated with different radiant exposures. The release efficiency was determined by the amount of cells per image with 3–50 cells showing relevant fluorescent change according to the chosen criteria. Results are similar to the decision by eye (samples without AuNPs and with non-CPP-AuNPs show this ratio). Spikes (maximal ratio: $\times$) are due to analysis. Threshold is found at 25 mJ/cm$^{2}$, efficient release for all higher radiant exposures. A two-tailed t-test revealed a significant increase compared to lower radiant exposures or control samples (***p $<$ 0.001, *p $<$ 0.04). *Box*: first to third quartile, *line*: median

For future clinical applications and to avoid harmful impact on the cells like vacuoles or blebbing (as described later) the applied radiant exposure should be kept to a minimum. Hence, we irradiated cells that were co-incubated with CPP-AuNPs and calcein for 4 h with different radiant exposures up to 35 mJ/cm$^{2}$. The obtained images were analyzed as described in the Methods section. The results are presented in Fig. 6. It shows the amount of cells with sufficient fluorescent changes per image according to the defined criteria (see “Methods” and Additional file 1: Section A5). Therefore indicating the probability for intracellular molecule release for the given parameters. Non-irradiated cells with endosomes containing CPP-conjugated AuNPs and calcein or cells irradiated with a radiant exposures of up to 20 mJ/cm$^{2}$ showed no significant calcein delivery into the cytoplasm. We found a release threshold at 25 mJ/cm$^{2}$. For this radiant exposure the amount of cells where the calcein was successfully delivered varies over the whole range. In some analyzed images nearly all cells showed an efficient calcein release while some images did not. For radiant exposures of 28 mJ/cm$^{2}$ and higher, the calcein was efficiently released on average in at least 58 % of the cells (Fig. 6, small solid boxes). We found a release in 81 % of the cells treated with 35 mJ/cm$^{2}$. A performed independent two-tailed t-test gave a statistical significant difference (p $<$ 0.001) for the release when applying radiant exposures $\ge$28 mJ/cm$^{2}$ compared to radiant exposures below the release threshold and all control samples.

As a control group, cells were also incubated without AuNPs or with non-CPP-conjugated particles which are not taken up (uptake studied in [47]) and irradiated with 35 mJ/cm$^{2}$. Their calcein release is comparable to non-irradiated cells containing CPP-conjugated particles inside endosomes. We attribute part of calcein uptake in the control groups to calcein settling on the cell membrane. Furthermore, some molecules can be taken up as the cells naturally endocytose.

### Cell viability and cytoxicity of AuNPs

Irradiation with radiant exposures of less than 100 mJ/cm$^{2}$ showed no visible cell damage under the microscope. Using radiant exposures of around 100–200 mJ/cm$^{2}$ or higher, the outer cell membrane was ruptured during the irradiation. This is indicated by the extracellular dye propidium iodide (PI) entering the cell. For even higher laser powers, severe and irreversible damages like instant necrosis and blebbing were induced (data not shown).

Quantitative analysis of the cell viability was performed as described in “Methods” by double staining the cells with PI and calcein AM (acetoxymethyl ester). This calcein derivate can pass the plasma membrane, but only fluoresces after the acetoxymethyl group is removed by intracellular esterases which are only active in live cells. Irradiating cells with up to 35 mJ/cm$^{2}$, which is enough to efficiently release molecules into the cytoplasm, has no significant effect on their viability. At least 94 % of the cells express calcein AM whereas only a maximum of 6 % of cells is PI positive which indicates cell death (Additional file 1: Figure A6). Cells expressing dual fluorescence were accounted as dead cells.

**Fig. 7:**
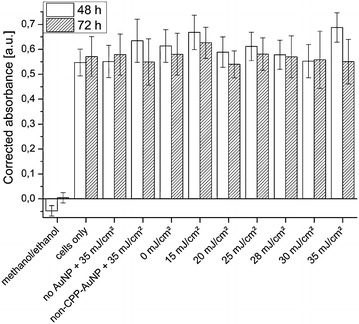
Metabolic activity of cells. Long-term influence on laser viability tested 48 and 72 h after laser treatment using PrestoBlue. All values are background corrected for the blank with only cell culture medium. Laser irradiation has no influence on cell proliferation. Cells killed with methanol/ethanol act as negative control. n = 6

We further checked the long-term viability of cells, more precisely their metabolic activity, using PrestoBlue (see “Methods”). For each set of parameters three different samples were evaluated. We did not find a difference in the metabolic activity of cells incubated with AuNPs conjugated with either peptide. Therefore, the results for the peptides CWR$_{10}$ and CWG$_{3}$PK$_{3}$RKVED were combined (n=6 for each data point). Overall, no reduced viability for the laser irradiated cells compared to non-irradiated cells was observed two and three days after irradiation (Fig. 7). For all parameters with living cells the absorption and, hence, the amount of cells, are in the same order of magnitude. The laser treatment also did not show an effect on the viability of cells incubated without particles or particles without peptide-conjugation. Solely the positive control with killed cells had a significantly lower metabolic activity.

## Discussion

Our TEM studies confirmed endocytosis as the uptake mechanism of the differently shaped and sized agglomerates of CPP-AuNPs which we suggested in our previous study [47]. Their hydrodynamic diameter perfectly suits the cellular uptake as the optimal diameter of spherical particles for receptor-mediated endocytosis is 54–60 nm [49]. AuNP-filled endosomes were found all over the cytoplasm. TEM-images showed that colloidal CPP-AuNPs are only loosely bound agglomerates but no aggregates with fixed solid-solid bridges. After being endocytosed they appeared to be arranged differently. Especially in late endosomes very condensed CPP-AuNPs without many extensions or cavities were found. We assume that the agglomeration to large clusters is rather flexible and can reshape according to the available space within the endosomes. TEM-images proved that the nanoparticles remain encapsulated during endosomal maturation. AuNPs condensing during the maturation process indicate that the biological material around the particles may be degraded. We ascribe the degradation to proteolysis as the concentration of proteases increases during the maturation [50]. To prevent the intraendosomal degradation and accumulation of the cargo it is necessary to externally trigger the endosomal opening and time the release accurately. Nonetheless, the release mechanism of the presented method will be unaffected by this as the nature of the endosomal membrane remains so that the effects induced by laser-particle interaction can rupture the membrane. We found, that the effects even rupture multi-lamellar membranes.

The exact influence of laser irradiation on CPP-AuNPs strongly depends on the size of the nanoparticle agglomerate and the radiant exposure applied. Ligand-free AuNPs have smaller extinction efficiency due to their smaller size and their surface plasmon resonance (SPR) peak is at wavelengths below 520 nm [51, 52]. Their conjugation leads to a red-shift and broadening of the SPR peak which increases with smaller interparticle distances and larger amounts of agglomerated AuNPs [53, 54]. Despite the shift, the extinction cross section and scattering efficiency increases at wavelengths around 532 nm while the absorption efficiency is reduced [53, 55]. Hence, the agglomerates possess an enhanced extinction cross section compared to individual AuNPs and yield hot spots which exceed the enhancement factor coming from the amount of AuNPs [55]. Even though larger wavelengths might be favorable for some agglomerates, we assume 532 nm to be suitable for our CPP-AuNPs as we only found a SPR peak shift of 20–40 nm. This is in good accordance with the hydrodynamic particle diameters measured. The SPR peak at 532 nm is maximal for particles with diameters of $\sim$48 nm [52].

As the intracellular release is based on the properties of the AuNPs and laser parameters their interaction is discussed before elaborating the impact of the resulting effects on the cell. We assume that the endosomal rupture is initiated by particle heating as we use rather long pulses of 1 ns and for particles with diameters smaller than 80 nm absorption dominates scattering [54]. Heat deposition in particles can lead to vibration of electrons and particle melting, evaporation or explosion [27, 28, 56, 57]. Our own calculations (not shown here) find maximal temperatures of the medium within the agglomerates slightly above the melting point of gold nanoparticles (1063 $^\circ$C) for the threshold radiant exposure of 25 mJ/cm$^2$. These temperatures lead to an explosive evaporation of the medium around the particles. Besides thermal damages to the endosomal membrane, the vapor pressure can be followed by a shock wave [27, 28]. As the association of AuNPs reduces the threshold to create bubbles also water or cavitation bubbles can be induced [25, 26]. Their expansion and collapse dynamics mechanically disrupts the endosomal membrane and releases the cargo [58]. Small variations in the agglomerate size did not influence the endosomal opening significantly, confirming the prevalence of thermal effects. In addition, our threshold of 25 mJ/cm$^{2}$ is similar to the 28 mJ/cm$^{2}$ calculated as particle evaporation threshold (55 nm AuNP in aqueous solution, $\lambda$ = 532 nm, $\tau_{pulse}$ = 5 ns) [57, 59].

It is sufficient to discuss single pulse interactions with the AuNPs as the distance of two pulses is 44.4 µs while the heat diffusion occurs within nanoseconds. Therefore, accumulative heat effects can be neglected. Furlani et al. found that 10 ns after the laser pulse the induced secondary bubbles have collapsed and 20 ns after the pulse the temperature will be close to the starting temperature again (for 60 nm particles) [60]. This is in good accordance with our calculations which found no significant residual heat 20 ns after irradiating the CPP-AuNPs with 25 mJ/cm$^2$. Additionally, our CPP-AuNP agglomerates burst when irradiated. Else than for a solid single particle of the same size as the agglomerate, a second laser pulse would not be able to induce the same effect again. Therefore, even after being released to the cytoplasm and maybe being irradiated again, no further damage can be induced to other cellular compartments.

Using a similar laser ($\lambda$ = 532 nm, $\tau_{pulse}$ = 0.5 ns) and 80 nm AuNPs in liposomes, Anderson et al. found plasmonic nanobubbles as small as 50 nm at a threshold of 110 mJ/cm$^{2}$ [38]. This agrees with the threshold of 100–200 mJ/cm$^{2}$ we found for collateral rupturing of the outer cell membrane during irradiation. This indicates that radiant exposures $>$200 mJ/cm$^{2}$ steadily induce large bubble formation leading to major cell damages. As a consequence direct blebbing occurred, affecting the cell viability. Thus, the radiant exposure defines the damage zone of our technique. Conjugation of CPPs to AuNPs allows precise subcellular targeting while still maintaining the cell integrity. The applied radiant exposures used for molecule release have no impact on cells without AuNPs.

Similar to the release using endosomolytic reagents our laser-based approach is temporally specific. Hence, our technique is applicable to overcome the problem that cargo is trapped in the endosomes and being degraded during their maturation [12, 61]. We conclude that laser light interacting with AuNPs is target selective as well as temporally specific. The occurring effects can be controlled by choosing both the laser and AuNP properties. Our active release circumvents intraendosomal cargo accumulation and slow penetration of the cargo to the cytosol.

The probability for cargo release increases with increasing radiant exposures as more energy is deposited. At a threshold radiant exposure of 25 mJ/cm$^{2}$ the molecule release probability is 50 %. It can be increased up to 81 % by applying 35 mJ/cm$^{2}$. The fluorescence intensity of non-targeted cells (without AuNPs, with AuNPs without CPPs) is less and TEM images show nearly no particles inside the cells (Additional file 1: Figure A4.3). The performed t-test clearly indicates a significant (p $<$ 0.001) increase of the ratio of cells with successful release for radiant exposures above the figured threshold and for all controls. The level of significance is less for the threshold itself (p $<$ 0.04 for radiant exposures underneath and controls) supporting 25 mJ/cm$^{2}$ as the measured threshold. Moreover, between 20 and 35 mJ/cm$^{2}$ we find a logarithmic increase of the release probability as a function of radiant exposure which is in good accordance to the results for the uptake of dye in optoinjection ($\lambda$ = 800 nm, $\tau_{pulse}$ = 130 fs) [62]. Hence, higher radiant exposures would only slightly increase the probability for molecule release and might strongly affect the cell viability. Conjugating the molecule directly to the AuNPs can further result in an even higher specificity. It would prevent the uptake of free floating extracellular molecules via normal endocytosis of non targeted cells.

Yet, not all intraendosomal dye molecules are released to the cytoplasm. This can be seen in fluorescent images still having bright fluorescent spots. We assume it is due to non, partially or transiently damaged endosomal membranes. More uniform AuNP-agglomerate sizes and shorter incubation times to ensure all endosomes in the same stage of maturation upon irradiation could increase the amount of molecules delivered.

Irradiation of the cells with $<$35 mJ/cm$^{2}$ and the presence of CPP-AuNPs had no effect on the cell viability and metabolic activity. As a result, we assume that cells irradiated with 35 mJ/cm$^{2}$ can cope with the small laser-induced vacuoles found on the TEM images that might be cytotoxic as vacuoles have an autophagic nature [63]. Nonetheless, we believe that significantly higher radiant exposures are likely to induced more effects that can lead to cell death.

After irradiation the agglomerates are released as primary particles ($\sim$5 nm) and isolated AuNPs disperse in the cytoplasm. We did not find any particles in the nucleus. This could be due to the fixation of the cells immediately after the treatment. Most of the particles have not had the possibility to diffuse. They were still found close to the endosome they escaped. Following results showing particles can enter the nucleus without regulations ($<$9 nm) or via interaction with the nuclear pore complex ($>$39 nm) [64], we expect our separated CPP-AuNPs to penetrate the nuclear envelope. It is also possible that the uptaken particles are exocytosed [3, 9, 16] after some time leading to a smaller amount of particles within the cells over time. After irradiation the exocytosis probability rises, as smaller nanoparticles exocytose faster [16] and therefore possible adverse effects to biological systems due to long-term exposure of AuNPs can be minimized. Additionally, the amount of AuNPs per cells may also decrease during proliferation. Further, elimination from the body is more likely for particles this size [65]. In addition, the reader should keep in mind that ligands coupled to inorganic nanoparticles may be subject to a natural biodegradation process. This can be deliberately included in the design of the agglomerates [41] but a recent study has also shown that even firmly attached polymer shells may be removed from the particles in vivo [66]. Naturally, agglomerates connected to CPPs could be subjected to a similar fate. Consequently, the transfer of the agglomerate uptake and photoinduced release of the endosomal content as well as the deagglomeration of agglomerates to in vivo environment would require a thorough examination of biodegradation, which, however, is beyond the scope of this experimental series.

Besides laser induced molecule delivery, our CPP-AuNPs can possibly be used as theranostic agents. We showed the possibility to efficiently deliver cell impermeable dyes. The same nanoparticles could be used to image the target cells before releasing the endosomal cargo. Depending on the imaging technique this could either be done by labeling particles to enhance their imaging qualities or by using pure nanoparticles. AuNPs in the size range of our agglomerates were shown to be quantifiable with confocal laser scanning microscopy [67]. Our technique offers the possibility to selectively release molecules only in cells chosen during the imaging. If contraindications for, e.g. the delivery of a drug are revealed, no laser irradiation is applied and no cargo is released. Here, the natural pH-change in the endosomes during their maturation could be utilized to dispose unwanted drugs as the molecules stay enclosed. On contrary, if findings indicate the necessity to induce cell death to the targeted cells, higher radiant exposures could easily be applied.

## Conclusions

Conjugating CPPs to AuNPs is an efficient tool to trigger the uptake of particles and molecules using a natural uptake mechanism and enhancing the release using the interaction of AuNP agglomerates and laser irradiation. This interaction directly disperses the agglomerates into highly biocompatible products. In detail we could show that these stable and defined peptide-conjugated gold nanoparticle agglomerates can be applied systematically for intracellular molecule delivery. The intraendosomal cargo can efficiently escape into the cytoplasm without affecting the cell viability. We demonstrate that this laser-triggered release is a fast, targeted and gentle method which can be applied to various cell types as the release-mechanism is cell-type independent. The spatial and temporal specific release facilitated by laser irradiation can circumvent intraendosomal content degradation. This may enhance the intracellular biological activity of the delivered cargo. Besides enabling the uptake of cell impermeable molecules, gold nanoparticle agglomerates attached to CPPs enhance the electromagnetic field of the incident laser light. This enhancement and heat deposition in the vicinity of the gold nanoparticles lead to a local rupture of the surrounding endosomal membrane. Thus, with our method, intraendosomal cargo can be efficiently released without impairing other cellular compartments. Besides the specificity of the release, the choice of CPPs or their combination with cell-specific binders may determine the selectivity of the delivery as well as the intracellular target while the release mechanism is unaffected. Particle deagglomeration shows the benefit of a faster AuNP removal from the cell and avoiding further damages to other compartments after being released to the cytoplasm if irradiated again. Hence, this technique may have future applications for pharmaceutical drug screening or basic gene therapy studies for medical research. In addition, the photo-induced deagglomeration results in a change of the optical properties of the agglomerates which could be utilized as a label-free indicator for successful endosomal rupture and molecular delivery.

## Methods

### Laser-based generation of gold nanoparticle-peptide agglomerates

Gold nanoparticle agglomerates were synthesized in a two-step process. PLAL was followed by an ex situ bioconjugation of the generated ligand-free gold nanoparticles with cell-penetrating peptides. This procedure enables a defined adjustment of the peptide to nanoparticle ratio and, hence, leads to defined particle-peptide agglomerates.

PLAL was done as described elsewhere [47] (see also Additional file 1: Section A1). After laser ablation the colloids possessed a bimodal size distribution still containing a significant portion of particles $>$10 nm. This larger particle fraction was removed by ultracentrifugation at 30,000*g* for 13 min, yielding totally monodisperse colloids with average diameters of 5 nm. These purified colloids were applied for all consecutive conjugation experiments and were furthermore utilized as a non-agglomerated control in all biological experiments.

Finally, the monodisperse AuNPs were separately bioconjugated with two different CPPs, deca-arginin (CWR$_{10}$) and a nuclear localization sequence (NLS, CWG$_{3}$PK$_{3}$RKVED). This ex situ bioconjugation was performed by mixing 40 µL of a 300 µM solution of the respective peptide with 4.46 mL of the gold colloid. The final gold mass concentration was 60 mg/L which corresponds to $\sim$29 peptide ligands per AuNP. Since nanoparticle agglomeration is induced by peptide conjugation and due to the fact that, once started, it is an ongoing process, it has to be stopped. Hence, bovine serum albumin (BSA) was added at a concentration of 2.5 g/L for stabilizing the agglomerates in order to prevent precipitation. BSA is suitable for stabilizing nanoparticles [68] as well as nanoparticle-peptide-agglomerates [47] which was demonstrated before. Additionally, it is biocompatible because it is already present in the cell culture medium by using fetal calf serum (FCS). Consequently, BSA is a stabilizer with optimum biocompatibility, keeping potential toxic cross effects to a minimum. After the preparation, samples were agitated for one hour by a vibratory shaker (Retsch, Germany).

The characterization of AuNP-CPP agglomerates was carried out via UV-Vis spectroscopy (Thermo Scientific evolution 201) and DLS (Malvern, Zetasizer Nano). For size determination 300 and 590 AuNPs were counted on TEM-images (Phillips CM 12, preparation see Additional file 1: A2). The resulting size distribution was fitted by a log-normal function.

### Cells and uptake of gold nanoparticles

For the experiments we used ZMTH3 cells which are derived from a canine pleomorphic mamma adenoma [69]. This cell line has been successfully applied for gold nanoparticle mediated laser manipulation before (e.g. [31, 35, 70]). Additionally, as ZMTH3 is a model cell line for human mamma adenoma the obtained results are likely to be transferable to human settings. These mammalian cells were cultured in RPMI-1640 (Rosewell Park Memorial Institute) cell culture medium with 10 % FCS and 1 % Penicillin/Streptomycin (all: Biochrom, Germany). Cells were incubated at 37 °C and 5 % CO$_{2}$. Depending on the experiments, they were either seeded in 35 mm glass bottom dishes or in 96-well plates with glass bottom a day before the experimental procedure.

As presented in [47], the peptide-conjugation of AuNPs leads to an endocytosis based uptake of the AuNP-conjugates into ZMTH3. It varies with time and AuNP-concentration without inducing cytotoxic effects. For the concentration of 2.46 mg/L, the uptake of CWR$_{10}$-conjugated AuNPs decreased after an incubation time of 4 h [47]. Hence, 4 h incubation with 2.46 mg/L of AuNPs was chosen for all experiments to obtain a large amount of intracellular nanoparticles using a moderate amount of AuNPs and a reasonable incubation time. 2.46 mg/L corresponds to $\sim$24 agglomerates/µm$^{2}$ cell growth area. For the same amount of agglomerates 3.0 mg/L NLS-AuNPs were used as NLS yields to slightly bigger agglomerates at the same peptide concentration. For the uptake the cells were incubated with the particles at 37 °C and 5 % CO$_{2}$. After incubation, the cells were washed twice with PBS to remove all remaining extracellular particle conjugates. Fresh culture medium was added before laser treatment.

For the TEM-studies the ZMTH3 cells were incubated with NLS-AuNPs. Directly after the experimental procedure they were prepared for TEM (see Additional file 1: Section A3). The obtained ultrathin sections were imaged with a CEM 902A TEM (Zeiss, Germany) and a 1k FastScan CCD-Camera (camera and software, Tietz Video and Image Processing Systems, Germany). Additionally, all TEM-images were contrast enhanced using ImageJ.

### Laser setup for intracellular molecule release

**Fig. 8:**
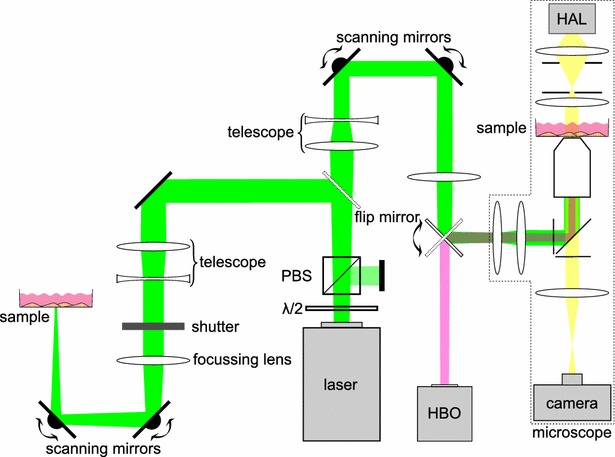
Setup for intracellular molecule release. Combination of half-wave plate ($\lambda$/2) and polarizing beam splitter cube (PBS) is used for power adjustments of the laser. The first flip mirror decides which setup the laser beam is guided to. *Left side* setup for high throughput release with large scanning areas. *Right side* Microscope-based setup for higher radiant exposures, but with a smaller scanning region

Two experimental setups were used to show the intracellular molecule release (Fig. 8). For both setups, a pulsed HLX-G-F020 microchip laser (Horus Laser, France) emitting 532 nm (22.5 kHz, $\tau_{pulse}$ = 1 ns) was used. For an immediate fluorescent visualization of the molecule release and cell membrane integrity, the laser was coupled into a microscope (Axiovert A.1, Zeiss, Germany) (Fig. 8, right side). To analyze the results, images were taken with a EMCCD-Camera (iXon DU-885, Andor Technology, UK) using the Andor Solis image acquisition software. The images were post-processed using ImageJ by enhancing their intensity and contrast as well as false color representation. High radiant exposures (8 J/cm$^{2}$) can be achieved with this setup. However, the irradiated area is limited due to the high focusing (50x objective, NA 0.5). To obtain molecule delivery in thousands of cells in a few minutes, the laser was coupled into another setup (Fig. 8, left side). As the laser beam is less focused, radiant exposures of up to only 35 mJ/cm$^{2}$ can be realized. This setup was used for parametric studies and statistical analysis of the delivery efficiency. In both setups the laser power is adjusted by combining a motorized half-wave plate with a polarizing beam splitter cube. Furthermore, both setups employ scanning mirrors to scan the whole region of interest meandering. The pulse to pulse distance in x-direction is 2.2 μm and 33.3 µm in y-direction.

### Properties and uptake of calcein

For the release studies, the cell impermeable fluorescent dye calcein was used as molecular cargo. Calcein was diluted to a final concentration of 0.5 mM in cell culture medium. For these studies the CWR$_{10}$-AuNPs were used.

To enter the cell, calcein was incubated with the AuNPs simultaneously. During the CPP-induced formation of endosomes to take up the CPP-AuNPs, medium containing calcein also enters the endosome. The cells were washed twice after incubation. Hence, all dye molecules in the extracellular medium were removed before laser irradiation. Hence, only the intraendosomal calcein is released into the cytoplasm.

### Image analysis to quantify the calcein release

To quantify the release of calcein, several objective criteria were established. Cells that took up the fluorescent calcein displayed fluorescing spots. After laser irradiation the fluorescent molecules were dispersed. We took fluorescent images (microscope setup) of cells before and after irradiation with different radiant exposures. For each parameter n $>$ 100 cells were analyzed. First, the decision on successful release was performed by eye for 1474 cells. Then, single cell analysis of the images was performed using ImageJ (detailed description in the Additional file 1: Section A5). By combining the evaluation by eye with the ImageJ analysis, we established two criteria for calcein release (Additional file 1: Figure A5.3). First, the fluorescent area per cell after laser treatment increased at least 5 % compared to before irradiation. Second, a minimum of 18 % of the cell area after irradiation fluoresces.

Per tested parameter these criteria were applied on 8–13 different field of views each showing 3–50 cells. Irradiation for all parameters was done on at least two different days. These images were used to calculate the ratio of cells per image showing an efficient release (according to the criteria).

### Viability assay

To account for the cell viability after the treatment, cells were detached using TrypLE (Life Technologies, USA). The cells were stained with fluorescent dyes for the analysis. We used 2.5 µM Propidium Iodide (PI, Life Technologies) to check for immediate cell death, i. e. mainly necrotic cells. At the same time we incubated the cells with 3 µM calcein AM (Life Technologies) to double check for the viability. After detaching, cells were spun down and added in a counting chamber. The Cellometer Vision 5$\times$ (Nexcelom Bioscience, USA) was used to count the total amount of cells as well as the amount of PI and calcein AM positive cells. For all parameters more than 3000 cells were counted (n $\ge$ 4).

To make sure the cell membrane is not compromised during the release, in some experiments PI was added into the extracellular medium before irradiation (in this case we did not check for necrotic cells using PI).

Furthermore, we studied the long-term influence (48, 72 h) of the laser treatment on the cell proliferation using 10 % PrestoBlue (Life Technologies) in cell culture medium. This experiment was performed in a 96 well plate with 1.5 $\times$ 10$^{4}$ cells/well. Therefore, the absorbance of the PrestoBlue was measured after an incubation time of 24 h (37 °C, 5 % CO$_{2}$) with a SPECTROstar Omega (BMG LABTECH, Germany) plate reader as recommended by the manufacturer. The measured absorbance was normalized using the emission at 600 nm as reference wavelength and background corrected by subtracting the value for only medium (according to the application note, PrestoBlue, Life Technologies). The resulting values represent the metabolic activity of the cells. As a negative control cells were killed directly after the treatment by incubating them for ten minutes with a 1:1 solution of Methanol (99.6 %) and Ethanol (99 %). The cell proliferation assay was performed for both CPP-AuNPs.

## Additional file

10.1186/s12951-015-0155-8 Detailed information on CPP-AuNPs, TEM, the release criteria and viability. The additional file includes a more detailed description of the particle synthesis, the preparation of the TEM samples and additional characteristics of CPP-AuNP agglomerates. Additionally, a gallery of further TEM-images as well as of primary AuNPs without CPPs and individualized AuNPs after irradiation are presented. Further, the ImageJ analysis including a selection of sample images to obtain the fluorescent area per cell before and after irradiation as well as the results of the cellular viability directly after laser treatment are shown.
