# Supplementary material for: Optical and electron microscopy study of laser-based intracellular molecule delivery using peptide-conjugated photodispersible gold nanoparticle agglomerates
Source: J Nanobiotechnology. 2016 Jan 8;14:2. doi: 10.1186/s12951-015-0155-8 (PMC4706709; doi:10.1186/s12951-015-0155-8)
Supplement: Supplementary file 1 — 10.1186/s12951-015-0155-8 Detailed information on CPP-AuNPs, TEM, the release criteria and viability. The additional file includes a more detailed description of the particle synthesis, the preparation of the TEM samples and additional characteristics of CPP-AuNP agglomerates. Additionally, a gallery of further TEM-images as well as of primary AuNPs without CPPs and individualized AuNPs after irradiation are presented. Further, the ImageJ analysis including a selection of sample images to obtain the fluorescent area per cell before and after irradiation as well as the results of the cellular viability directly after laser treatment are shown. [file 12951_2015_155_MOESM1_ESM.pdf]

ADDITIONAL INFORMATION

Optical and electron microscopic study of laser-based intracellular molecule delivery using peptide-conjugated gold nanoparticles

Judith Krawinkel[1*], Undine Richter[1], Maria Leilani Torres-Mapa[2], Martin Westermann[3], Lisa Gamrad[4], Christoph Rehbock[4], Stephan Barcikowski[4] and Alexander Heisterkamp[2,5]

Detailed information on CPP-AuNPs, TEM, the release criteria and viability
The additional file includes a more detailed description of the particle synthesis, the preparation of the TEM samples and additional characteristics of CPP-AuNP agglomerates. Additionally, a gallery of further TEM-images as well as of primary AuNPs without CPPs and individualized AuNPs after irradiation are presented. Further, the ImageJ analysis including a selection of sample images to obtain the fluorescent area per cell before and after irradiation as well as the results of the cellular viability directly after laser treatment are shown.

*Correspondence: judith.krawinkel@uni-jena.de
[1]Institute of Applied Optics, Friedrich-Schiller-University Jena, Fröbelstieg 1, 07743 Jena, Germany
Full list of author information is available at the end of the article

A1 Detailed description of the particle synthesis

For pulsed laser ablation in liquid (PLAL) we used a picosecond laser (Ekspla Atlantic) with a pulse duration of 10 ps and a repetition rate of 100 kHz at a wavelength of $\lambda = 1064$ nm. The pulse energy behind the scanner optics was 110 µJ. The laser beam was focused through a 3 mm liquid layer on a gold target of 500 µm thickness. The target was fixed on a holder and placed in a 30 mL batch chamber applying a lens with a focal length of 100 mm. For ablation the laser beam was scanned with 6 mm/s along a spiral pattern with an external diameter of 6 mm for 10 minutes. The ablation liquid consisted of a sodium phosphate buffer (NaPB) at a salinity of 600 µM at pH 8.

A2 Additional characteristics of CPP-AuNP agglomerates

The ligand-free AuNPs, the primary particles of the agglomerates, have a hydrodynamic particle diameter of (5.0 ± 0.6) nm even though the exact size varies with the measuring technique used (Figure A2.1). The hydrodynamic particle diameter for the deca-arginine AuNPs is smaller (Figure A2.2) compared to NLS-AuNPs. Therefore they yield to smaller agglomerates at the same peptide concentration. Furthermore, a shift of the surface plasmon resonance of 20 nm for CWR_{10}-AuNP agglomerates and 40 nm for NLS-AuNP agglomerates can be observed compared to ligand-free AuNPs (Figure A2.3). The zeta potential of these agglomerates is $(+25.9 \pm 5.0)$ mV as compared to $(+18.7 \pm 14.9)$ mV for NLS-AuNPs (Figure A2.4). Further characteristics of the CPP-AuNP agglomerates were published by [1].

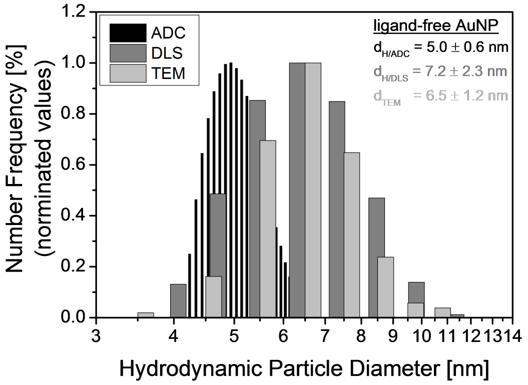

Figure A2.1: Ligand-free AuNPs have a hydrodynamic particle diameter of (5.0 ± 0.6) nm when determining the size distribution using an analytic disc centrifuge (ADC). In DLS and TEM the measured diameters are slightly larger.

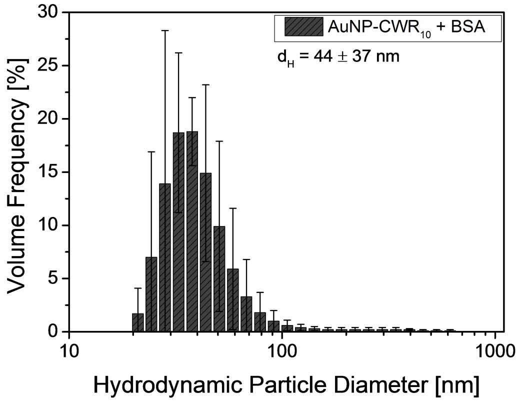

Figure A2.2: After agglomeration CWR_{10}-AuNP agglomerates have a hydrodynamic particle diameter (Volume Frequency) of (44 ± 37) nm.

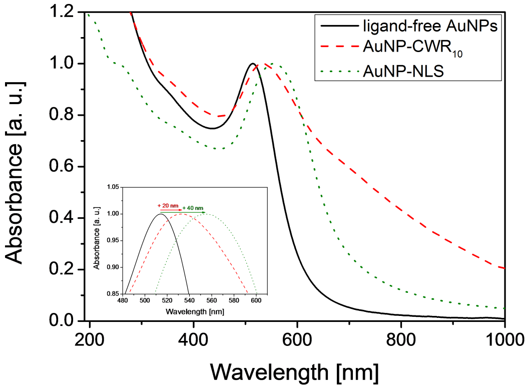

Figure A2.3: Comparison of the surface plasmon resonance peak of ligand-free AuNPs and the agglomerates. CWR_{10}-AuNP agglomerates show a shift of 20 nm, NLS-AuNP agglomerates a shift of 40 nm. The peak broadens for the agglomerates and thus, the shift is larger for longer wavelengths.

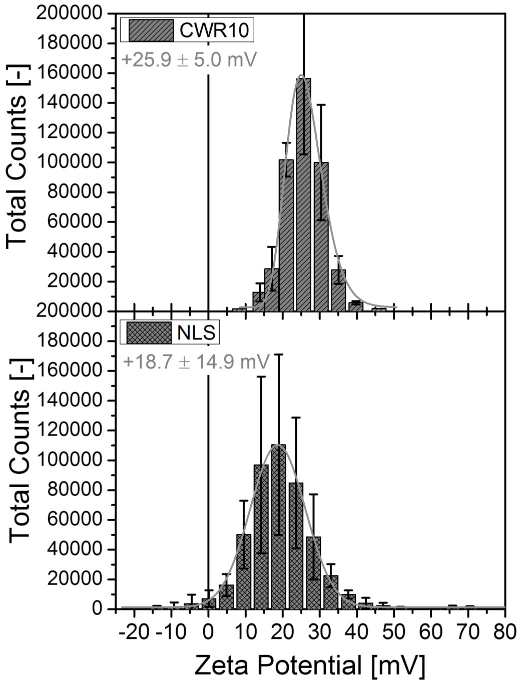

Figure A2.4: The zeta potential of the CWR_{10}-AuNP agglomerates is $(+25.9 \pm 5.0)$ mV compared to $(+18.7 \pm 14.9)$ mV for NLS-AuNP agglomerates.

A3 TEM

Particles were prepared for TEM imaging following the negative staining method of Nermut [2]. The particle suspension was diluted 1:10 in twice distilled water (except for AuNPs without BSA). A droplet was adsorbed to a 400-mesh copper grid coated with formvar-carbon (Quantifoil, Germany). Following, the sample was stained with 2 % uranyl acetate in twice distilled water.

For the cell samples, ZMTH3 cells were prepared and incubated with CWG_3PK_3R-KVED-AuNPs (see methods subsection: Cells and uptake of gold nanoparticles). After the experimental procedure, cells were directly prepared for transmission electron microscopy (TEM). First, cells were fixed with 2.5 % glutaraldehyde in PBS for 60 minutes at room temperature. After washing with PBS and cacodylate buffer they were scraped off from the dishes and sedimented. Cells were postfixed for 120 minutes with 1 % osmium tetroxide gradually dehydrated in a series of ethanol solutions and embedded in Araldite resign (Agar Scientific, England) [3]. Ultrathin sections of about 70 nm thickness were cut with an Ultramicrotom (Ultracut E, Reichert-Jung, Austria) and mounted on 100-mesh formvar-carbon coated copper grids (Quantifoil, Germany). The ultrathin sections were imaged with a CEM 902A transmission electron microscope (Zeiss, Germany) and a 1k FastScan CCD-Camera (camera and software, Tietz Video and Image Processing Systems, Germany). Additionally, all TEM-images were contrast enhanced using ImageJ.

In addition to figure 4, a variety of TEM images of cells containing CPP-AuNP agglomerates after irradiation with 25 mJ/cm^2 and 35 mJ/cm^2 are shown in figure A3.1 and figure A3.2, respectively.

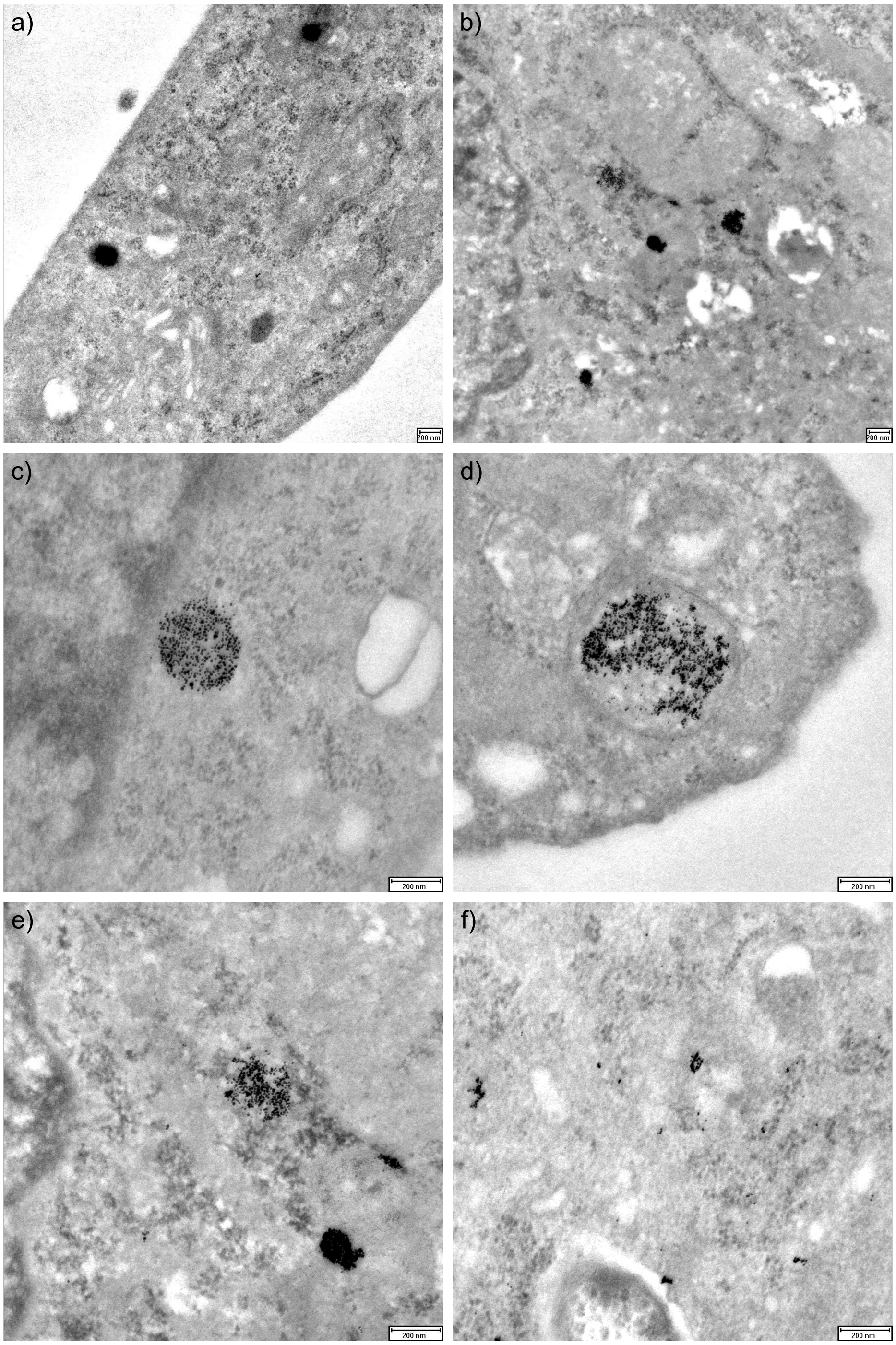

Figure A3.1: ZMTH3 with intraendosomal NLS-AuNP agglomerates after irradiation with 25 mJ/cm^2. All scale bars: 200 nm.

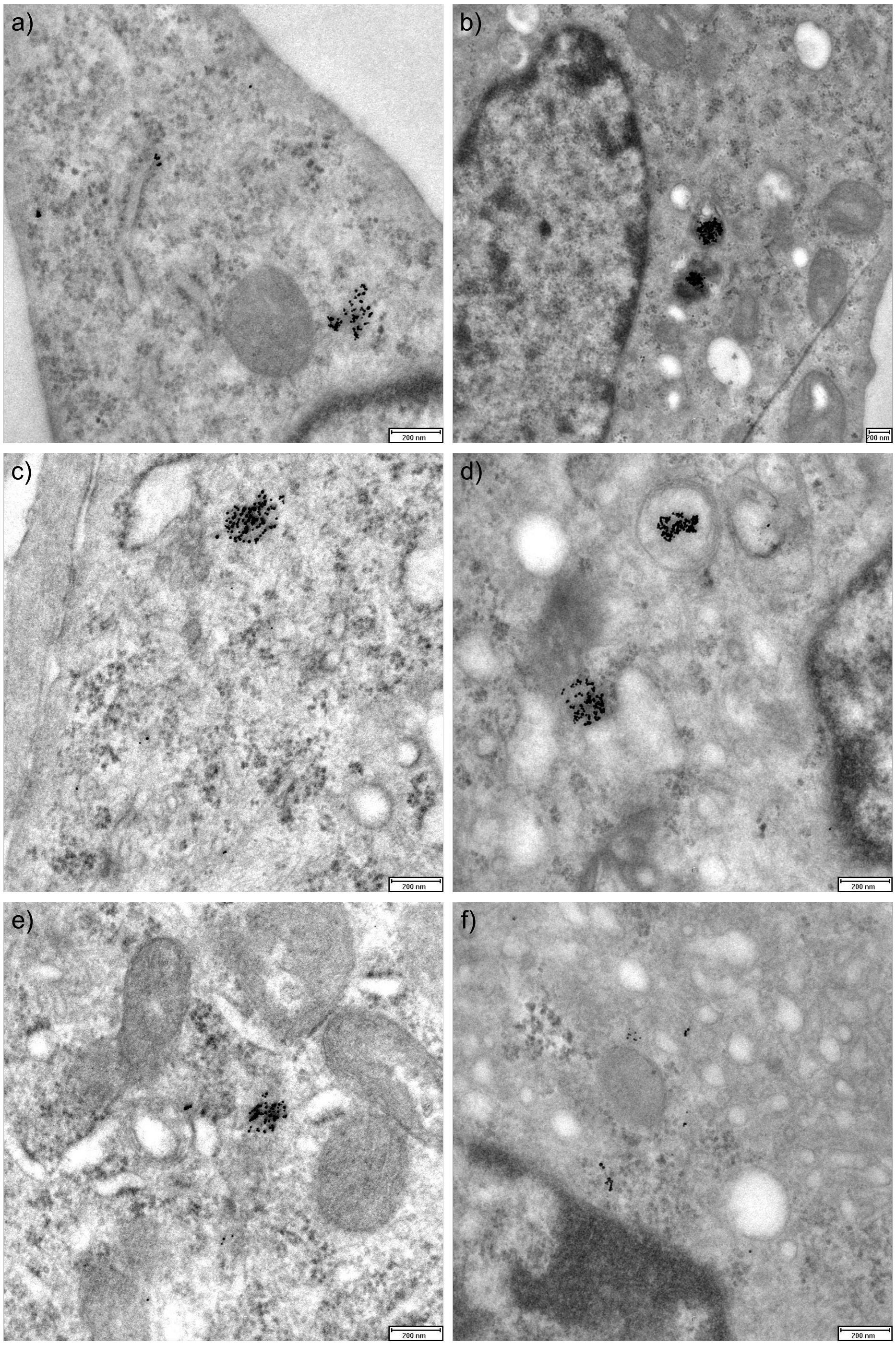

Figure A3.2: ZMTH3 with intraendosomal NLS-AuNP agglomerates after irradiation with 35 mJ/cm^2. All scale bars: 200 nm.

A4 Isolated particles

After irradiation of the agglomerates with 35 mJ/cm^2 a non-electron dense BSA-sheath having a similar shape to the agglomerates with particle leftovers could be found (Figure A4.1).

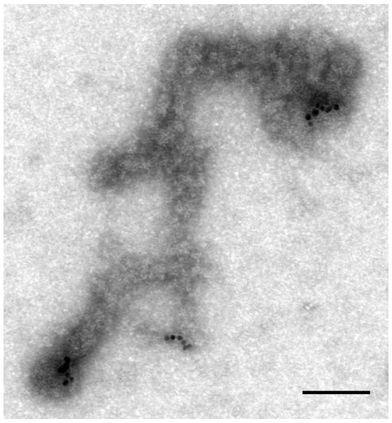

Figure A4.1: Exemplary TEM image of a BSA-sheath after irradiation with 35 mJ/cm^2 in the shape of an agglomerate with leftover particles only. Scale bar: 100 nm.

Irradiating CPP-AuNPs leads to a desagglomeration of the particles into individual particles or small particle clusters (Figure A4.2b) similar to non-CPP-AuNPs (Figure A4.2a).

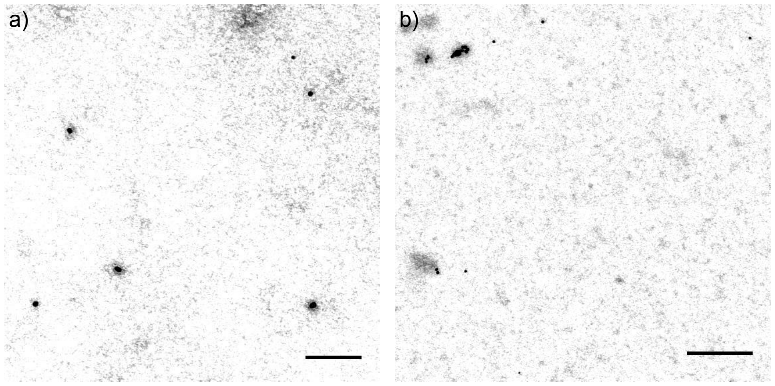

Figure A4.2: Exemplary TEM images of a) non-CPP-AuNPs and b) CPP-AuNP irradiated 35 mJ/cm^2. Scale bars: a) 100 nm, b) 200 nm.

Non-CPP-AuNPs are not efficiently taken up by the cells. Only individual particles can be found sporadically inside the cells (Figure A4.3)

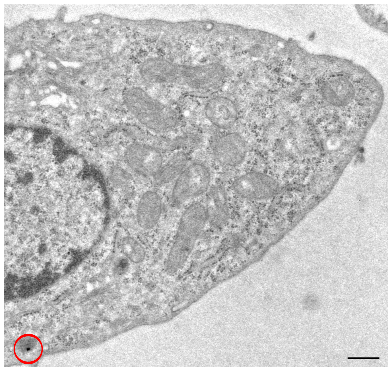

Figure A4.3: Exemplary TEM image of a cell incubated for 4 h with non-CPP-AuNPs. Only sporadically individual AuNPs can be found inside the cell (red circle). Scale bar: 500 nm.

A5 ImageJ analysis to obtain fluorescent area per cell before and after irradiation to define criteria for the release

Exemplary images of cells that were analyzed are shown in figures A5.1 and A5.2. Each image shows a field of view before or after irradiation with 25 mJ/cm^2 and 35 mJ/cm^2 respectively. The cells in the according images after irradiation marked red were counted as not released, the ones circled yellow showed successful calcein release according to the criteria defined in more detail below.

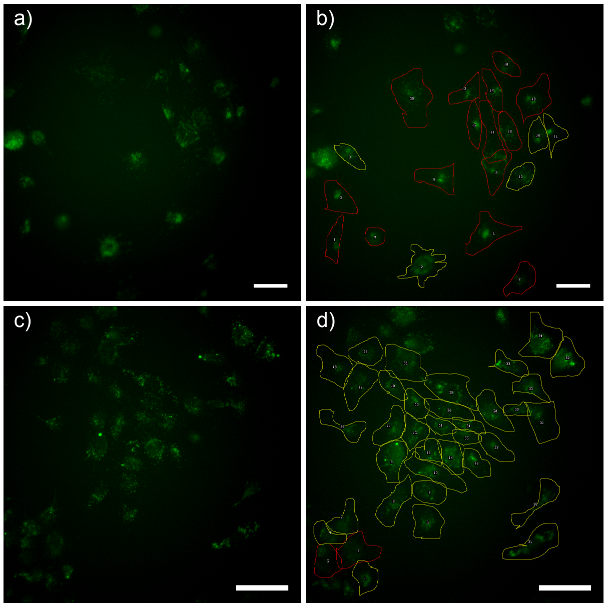

Figure A5.1: Two different fields of view evaluated before (a, c) and the according images after irradiation with 25 mJ/cm^2 (b, d). Yellow circled: cells successfully released calcein. Red circled: no successful release. All scale bars: 50 µm.

Each cell area was selected by defining a region of interest (ROI) with the help of the ImageJ Livewire plug-in. A user-written macro applying the ImageJ analysis

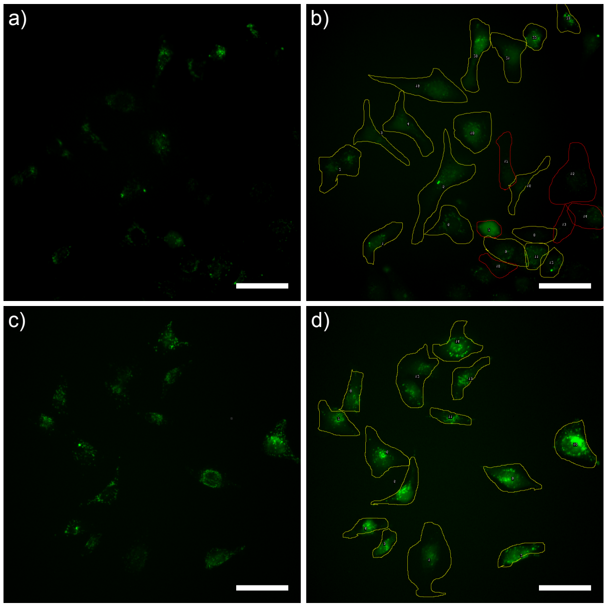

Figure A5.2: Two different fields of view evaluated before (a, c) and the according images after irradiation with 35 mJ/cm^2 (b, d). Yellow circled: cells successfully released calcein. Red circled: no successful release. All scale bars: 50 µm.

tool “measure” calculated the area of the ROI. A background (mean gray value of a chosen region next to the ROI of every cell) was subtracted. Applying a threshold of 10 % above this background when converting the ROIs into binary images displays only pixel with an intensity above this threshold. Calculating the amount of pixels results in the fluorescent area of a cell. To choose criteria for an effective molecule release the percentage of the fluorescing area of every cell after irradiation is plotted against the ratio of the fluorescing area after and before laser irradiation (Figure A5.3). Each diamond represents one cell. Blue diamonds are cells that showed a successful molecule release examining the ROIs by eye. No release was observed for the cells represented by pink diamonds, meaning that for these cells the fluorescent spots (which are endosomes with CPP-AuNP agglomerates and calcein) were still visible (as they were before the irradiation) and no fluorescence could be observed outside the endosomes, i.e. inside the cytoplasm.

With these observations, objective criteria for a successful release were determined as follows. Cells showed successful calcein release when their fluorescent area was at least 18 % of the total area and a minimum of 5 % larger than before irradiation (i.e. a ratio of 1.05 of the fluorescent cell area after to before irradiation). With these criteria, 83 % of the cells that were labeled as “molecule released” with the analysis by eye were picked. Furthermore, 14 % of the cells were false positive, meaning that no release was detected by eye, but the cells were counted using the chosen criteria. This compensated the percentage of cells, that were counted as “molecule released” by eye but not when using the criteria. Therefore, the chosen criteria can be applied. Some cells have a smaller fluorescent area after the treatment. This is mainly due to bleaching effects and very faint fluorescence. Furthermore the background is not constant over the images. The mean fluorescence intensity over the whole cell

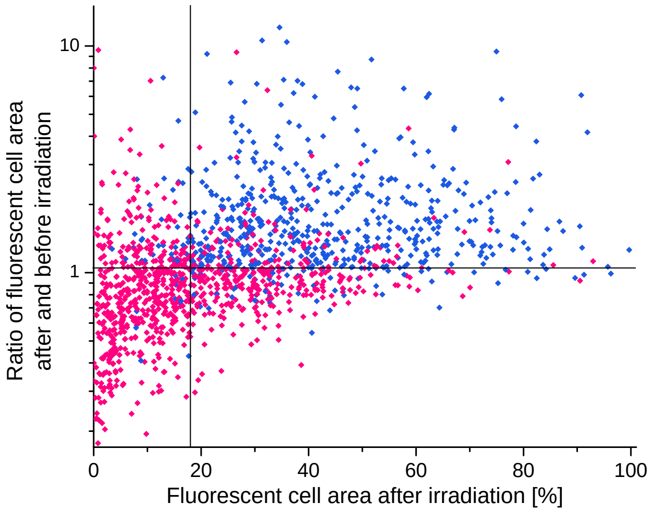

Figure A5.3: Percentage of fluorescent area of 1474 cells (for all parameters) after the treatment against the ratio of their fluorescent area after and before irradiation (logarithmic representation). Blue diamonds indicate cells that showed an efficient molecule release into the cytoplasm by eye. Pink diamonds represent cells we could not detect the release by eye. The black lines display the best possible criteria to choose cells with successful molecule release using ImageJ (upper right quadrant).

before and after irradiation could not be used as a criteria as the difference is not significant. This can be explained as the amount of fluorescent molecules in the cell is not changing with irradiation, only their distribution within the cells is altered. Nonetheless, the fluorescent intensity is not uniform over the whole cell as, e.g., there is no calcein in the nucleus of the cells and, hence, due to the threshold applied to obtain the binary images, not the whole cell area is fluorescent. The need to define one criteria considering only the fluorescent area after irradiation is attributed to the intense, but highly localized fluorescence which cells without a successful calcein release still have and which accounts for up to 18% of the cell area (but not for all cells).

Parts of the detected fluorescence change can also be attributed to the image analysis. Outliers in Figure 6 occur because of the strong changes in the background fluorescence. Similar observations were made for cells incubated without AuNPs. For this sample we found a distinct difference for the evaluation of the release ratio by eye compared to using ImageJ (in average 19 % compared to 32 %). Thus, it is difficult to apply the established criteria on these two controls as their uptake is different. Therefore we used the image analysis by eye to calculate the release efficiency for these.

A6 Viability of cells after laser treatment

Directly after the laser treatment we checked the cell viability using a double staining with calcein AM for viable cells and Propidium Iodide for dead, i. e. necrotic cells.

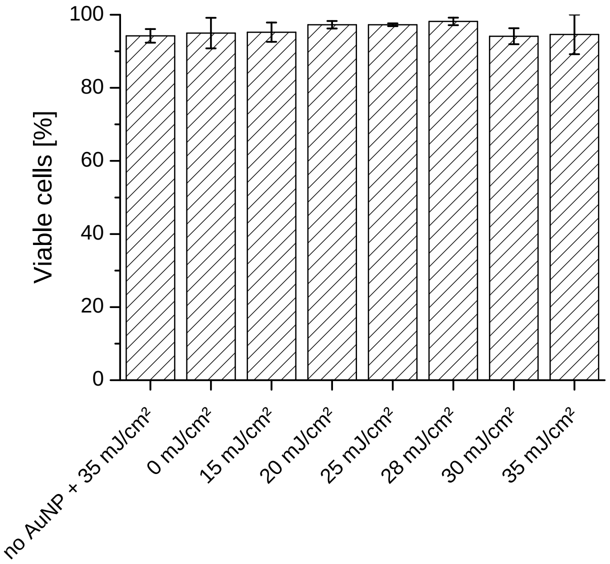

Figure A6: Viability of cells about ten minutes after the laser treatment. Stained with calcein AM (viable cells) and Propidium Iodide (dead cells). Counted automatically with the Cellometer. Dual (calcein AM and PI) fluorescing cells were accounted as dead. Non fluorescing, counted cells were subtracted from the total amount of cells as they were no cells but rather debris. More than 3000 cells were evaluated for all parameters ($n \geq 4$). No significant decrease for higher radiant exposures.

Author details

[1]Institute of Applied Optics, Friedrich-Schiller-University Jena, Fröbelstieg 1, 07743 Jena, Germany. [2]Institute of Quantum Optics, Leibniz University Hannover, Welfengarten 1, 30167 Hannover, Germany. [3]Electron Microscopy Centre, Friedrich-Schiller-University Jena, Ziegelmühlenweg 1, 07743 Jena, Germany. [4]Technical Chemistry I, University of Duisburg-Essen and Center for NanoIntegration Duisburg-Essen CENIDE, Universitätsstraße 7, 45141 Essen, Germany. [5]Excellence Cluster REBIRTH, Hannover, Germany.

References

1. Gamrad, L., Rehbock, C., Krawinkel, J., Tumursukh, B., Heisterkamp, A., Barcikowski, S.: Charge Balancing of Model Gold-Nanoparticle-Peptide Conjugates Controlled by the Peptide's Net Charge and the Ligand to Nanoparticle Ratio. J. Phys. Chem. C **118**(19), 10302–10313 (2014). doi:10.1021/jp501489t
2. Nermut, M.V.: Negative staining of viruses. J. Microsc. **96**(3), 351–362 (1972). doi:10.1111/j.1365-2818.1972.tb01064.x
3. Glauert, A.M., Glauert, R.H.: Araldite as an embedding medium for electron microscopy. J. Biophys. Biochem. Cytol. **4**(2), 191–194 (1958)
